# Health system capacity: maternal health policy implementation in the state of Gujarat, India

**DOI:** 10.3402/gha.v6i0.19629

**Published:** 2013-03-22

**Authors:** Linda Sanneving, Asli Kulane, Aditi Iyer, Bengt Ahgren

**Affiliations:** 1 Division of Global Health (IHCAR), Department of Public Health, Karolinska Institutet, Solna, Sweden; 2 Centre for Public Policy, Indian Institute of Management Bangalore, India; 3 Nordic School of Public Health, Gothenburg, Sweden

**Keywords:** maternal health, policy, implementation, India, Gujarat, health system capacity

## Abstract

**Introduction:**

The Government of Gujarat has for the past couple of decades continuously initiated several interventions to improve access to care for pregnant and delivering women within the state. Data from the last District Family Heath survey in Gujarat in 2007–2008 show that 56.4% of women had institutional deliveries and 71.5% had at least one antenatal check-up, indicating that challenges remain in increasing use of and access to maternal health care services.

**Objective:**

To explore the perceptions of high-level stakeholders on the process of implementing maternal health interventions in Gujarat.

**Method:**

Using the policy triangle framework developed by Walt and Gilson, the process of implementation was approached using in-depth interviews and qualitative content analysis.

**Result:**

Based on the analysis, three themes were developed: lack of continuity; the complexity of coordination; and lack of confidence and underutilization of the monitoring system. The findings suggest that decisions made and actions advocated and taken are more dependent on individual actors than on sustainable structures. The findings also indicate that the context in which interventions are implemented is challenged in terms of weak coordination and monitoring systems that are not used to evaluate and develop interventions on maternal health.

**Conclusions:**

The implementation of interventions on maternal health is dependent on the capacity of the health system to implement evidence-based policies. The capacity of the health system in Gujarat to facilitate implementation of maternal health interventions needs to be improved, both in terms of the role of actors and in terms of structures and processes.

Globally, the maternal mortality rate (MMR) fell from 400 deaths per 100,000 live births in 1980 to 260 in 2008 (1, 2). The progress achieved is, however, unevenly distributed between regions in the world and between nations. Maternal mortality is a health concern greatly affected by the gap between what is known in terms of medical interventions needed to prevent deaths and the services reaching pregnant and delivering women. Around the world women are dying during pregnancy, delivery, and the postnatal period not because lack of knowledge on how to prevent and treat these complications, but because of limitations in health systems and structures in society preventing women from having access to health care (3–6). To address this, the focus of interventions has been on the technical aspect such as facilitating emergency obstetric care, the structure of delivery care such as the availability of skilled attendants at birth, and on social interventions such as preventing adolescent pregnancy (7, 8). Increasingly, however, it is recognized that improved maternal health is dependent on the functioning of entire heath systems rather than on single interventions (9, 10). The multifaceted nature of maternal health demands a wide range of complex and comprehensive interventions to achieve progress in reduction of maternal mortality (3). The complexity comprises several possible complications and it is partly due to each of these complications being dependent on several single interventions to prevent death. This means that the effectiveness of a policy with the objective of improving maternal and reproductive health is dependent on the effectiveness of a package of interventions. It also means that the objective of the policy is highly dependent on each intervention to be achieved. This makes maternal health greatly dependent on the capacity of health systems.

The setting for this study is the state of Gujarat, located in the west of India. Gujarat has a population of just above 60 billion and is a highly urbanized state with 42% of the population living in cities (11). Gujarat has experienced rapid economic growth over the last decade, and the Government of Gujarat has for the past couple of decades continuously initiated several interventions to improve access to care for pregnant and delivering women within the state (12). Despite comprehensive measures to improve the situation, challenges to implementation remain (13–15). The objective of the study presented in this paper was to explore the process of implementing policies aiming at improving maternal health in the state of Gujarat.

## Conceptual framework

It is becoming increasingly acknowledged that knowing what is needed and facilitating these needs are not the same as ensuring improved health (16). The academic literature on studies conducted on health policy processes in low- and middle-income settings is scarce, and the focus of those conducted is often on ‘what happened’ rather than asking questions as to ‘what explains what happened’ (9).

In Gujarat, studies are available that describe the situation in terms of use of and access to maternal health care services. Data are available on coverage of interventions such as antenatal care and institutional delivery (8), and studies have provided insights as to what is needed in terms of, for example, human resources, equipment, supplies, and routines (17–19). By focusing on the process of implementation, this study attempted to explore possible explanations that go beyond describing ‘what happened’. The conceptual framework developed for this study serves as a foundation to frame and analyze questions related to exploring a deeper understanding of the process of implementing maternal health in the context of Gujarat.

### Policy analysis

There are several definitions of a *public policy* and several models developed on how to understand the role of *implementation* in the policy process. This study has used a model of the policy cycle developed by DeLeon and Brewer, which recognizes that a policy process is an ongoing cycle where different stages are closely interlinked with each other and that the context influences both the development and implementation of policy (20). The implementation is considered as a stage in the policy cycle, which means that implementation is closely interlinked with other stages such as agenda-setting and policy-making.

**Fig. 1:**
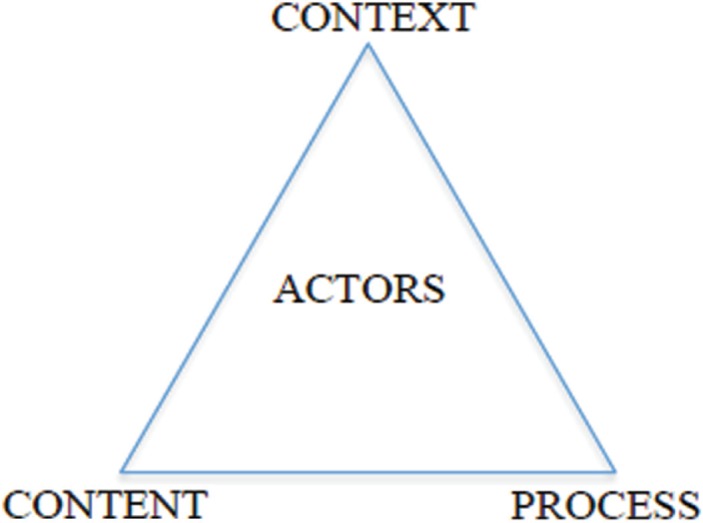
The policy triangle – a model of policy analysis (23).

The *policy triangle framework*, developed by Walt and Gilson, is used to provide a framework for how to study the policy process (21) (Fig. 1). This framework offers a model of policy analysis that captures some of the comprehensiveness of the policy process. The framework stresses the importance of going beyond the content of a policy when studying the policy process and emphasizes the importance of actors, the processes, and the context. The policy triangle considers how these aspects of policy interact and shape the policy process. The objective of this study is to explore the perception of the different aspects of implementation among actors involved in the process, using the policy triangle as a tool to assess the policy process.

## Methodology

### The setting

India accounts for a large proportion of the annual deaths resulting from pregnancy-related complications (22). In India, many women lack access to adequate maternal health care and many women continue to give birth at home (23). Large differences in the use of and access to maternal health care services exist between states within India; MMR is estimated to be as high as 390 in Assam compared to 81 in Kerala (24). Large differences also exist between different sub-populations within states, where economically and socially disadvantaged women are most vulnerable to pregnancy-related complications (25). Preconditions and challenges to progress towards improved maternal health are likely to differ between states, and progress is likely to be dependent on the implementation of context specific policies and interventions.

Gujarat is one of the most progressive states in India, with rapid socioeconomic growth. According to the latest District Level Household and Facility Survey (DLHS 3) conducted in Gujarat in 2007–2008, 56.4% of the women had institutional deliveries and 71.5% received at least one antenatal check-up (8). However, the coverage of maternal health care services is unevenly distributed between regions and sub-populations. In Gujarat, the health system challenges to improved maternal healthcare (e.g. lack of health professionals, weak referral systems, and lack of facilities and supplies) are well known, and interventions have been developed to meet these challenges. The training of doctors in gynecology and anesthesia and training of midwifes is ongoing, the infrastructure is being improved to ensure functioning referral systems, and facilities are being upgraded to so-called First Referral Units where basic and comprehensive emergency obstetric care should be available – these are all examples of activities that are ongoing in order to strengthen the capacity of the health system to provide maternal healthcare (26–28). Despite these efforts, the persistence of maternal mortality and morbidity remains one of the most pressing public health concerns in Gujarat.

### Pilot study

To test the feasibility of having a broad study objective, a pilot study was conducted in 2008. The pilot study tested the feasibility of asking open-ended questions on the topic of implementation of maternal health interventions in Gujarat. The use of probing questions was also tested. The information collected during the pilot study was used to develop the interview guide and to identify stakeholders relevant to explore the objectives of this study. Interviews and the stakeholders interviewed in the pilot study were not included in the study presented here.

#### Data collection

The process of identifying stakeholders for the study was guided by the conceptual framework and by the findings from the pilot study. Using DeLeons and Brewers model of a policy cycle, the implementation was regarded as an integrated part of the policy process rather than a solely administrative task, and this was reflected in the inclusion of stakeholders. Altogether, 12 high-level stakeholders with extensive experience from working with maternal health in Gujarat were interviewed. Roughly speaking, the stakeholders can be divided into three groups: those that work with or close to politics that influence maternal health policy, those that work with designing the interventions, and those that work with the management of implementing the interventions into the heath system. Obviously, these three areas of responsibility overlap and several of the stakeholders had experience from more than one, especially those stakeholders working with design and implementation overlap. Some of the stakeholders also have experience working with non-governmental organizations in the field of maternal health. Each key stakeholder was interviewed individually. The interviews lasted about 90 min, and each interview took place at a location chosen by the interviewee. The interviews were conducted in English, tape recorded, and transcribed verbatim. They were carried out in the fall of 2009 and the spring 2010.

The conceptual framework was also used when designing the interview guide. Following the model of the policy triangle, open-ended questions and probing questions were asked in relation to the *content* of the interventions, the *context* in which they are being implemented, the *actors* involved in the process, and on the structures of the *process*. The interview guide was not designed to directly ask about these aspects of implementation in separate questions. The ambition was rather to let the respondents speak freely on matters relevant for the objective of this study and for the interviewer to probe on these aspects of the policy process.

#### Qualitative content analysis

To analyze the transcribed interviews, qualitative content analysis was used as defined by Hsieh and Shannon: ‘a research method for the subjective interpretation of the content of text data through the systematic classification process of coding and identifying themes or patterns’ (29). *Conventional content analysis* was used to approach the data, which means that the use of preconceived categories was avoided and that the codes and categories are derived directly from the text rather than being based in a specific theory (29). The first author carried out the analysis. The conceptual framework was used in the design the study; however, the conceptual framework was not used as a model to organize or analyze the data. The analysis involved constantly moving between the entire data set, individual interviews, and coded extracts from the data. The process of analysis can be described as recursive rather than linear, where steps of analysis are used to structure the analysis but with movement back and forth between. The steps taken in the analysis were: 1) reading the entire data set repeatedly to obtain a sense of the depth of the data, 2) coding each transcript individually, 3) developing themes based on codes, 4) reviewing the themes by going back to the text and extracting codes, and 5) defining and naming the themes. Coding here is referred to as the process of organizing the data by extracting text relevant for the study objective and by giving the text a code. The development of themes was conducted by looking at the relationship between the codes and through interpretation of patterns found throughout the data set. However, it is important to stress that these steps overlap and that the analysis was a process that meant moving back and forth between these.

## Results

Based on the analysis, three themes were developed: lack of continuity; the complexity of coordination; and lack of confidence and underutilization of the monitoring system. Each theme is presented individually below.

### Lack of continuity

The objectives on maternal health under the framework of the National Rural Health Mission (NRHM), increased institutional deliveries and increased access to basic and comprehensive obstetric care, are in Gujarat being facilitated through the implementation of several interventions. Development of these interventions and the implementation plan for each intervention is a responsibility of the Government of Gujarat. Findings from the interviews show that the work on maternal healthcare at the state level in Gujarat is more dependent on individual stakeholders than on sustainable structures and processes. One respondent explains this as

everyone wants to come up with innovative ideas, no one wants to evaluate, monitor and coordinate existing policies. So all our funds are put into some new project, some new innovation. Going back to square one, I perceive, is the need of the day. We should find out the strength and weaknesses of the various processes’. Another respondent says in line with this that ‘eventually the heads change, the view change …. without evaluating the work we have done we are asked to go back.

The dependency of individuals rather than on structures and processes is perceived by the respondents to have an impact on long-term objectives and long-term planning. With weak structures and poorly established processes, the long-term memory and lessons learnt are lost when there is a shift in heads.

There are no stable policies in our health care system and the reason behind is that if policies are to be stable, they need to be framed, evaluated, modified and continued for care …. when a new body or person comes and looks at the same problem by a different angle, again new policies come and old policies are forgotten. What is missing is continuum of care.

Findings from the interviews show that the respondents perceive the lack of evaluation and follow-up as a barrier to improved implementation. Yet, variation as to how extensive this problem is in the setting of Gujarat varies between the respondents.

### The complexity of coordination

The responsibility and accountability of maternal healthcare in Gujarat is divided between several departments and divisions within the Government of Gujarat. Over the last decade, the power over health-related issues has also been decentralized to local governmental bodies called Panchayats. Additionally, the involvement of private providers and NGOs in providing maternal healthcare services is widespread throughout the state. This creates a comprehensive and complex network of institutions and stakeholders that are involved in developing, implementing, and administrating maternal healthcare programs and interventions. The combination of this complex network and the comprehensive number of interventions required to provide maternal healthcare requires tight coordination. The evaluation of the first phase of the Reproductive and Child Health Programme conducted by the World Bank stressed weak capacity to coordinate the program as a key challenge to its implementation. Improvements in coordination have been completed in the second phase of the program, and as a part of the strategy of the NRHM. However, the results from the interviews of this study highlight concerns about the limitations in coordination still remaining and that this should be considered a challenge to improved implementation of maternal healthcare interventions.

#### The impact of actors and structures in coordination

The results from the interviews show that coordination of maternal health interventions is acknowledged as an important aspect of improving the health system to provide comprehensive maternal healthcare. Coordination between actors at different levels of the health system within specific interventions was brought up during the interview. However, coordination between interventions and at system level was the focus of most interviews. One respondent puts it as:

If one component of the system fails the entire system fails.

The need for structures and processes that facilitate a platform to establish an overview and technical capacity to develop and implement interventions on maternal healthcare is brought up as a challenge to improved health system capacity.

A strong health resource center is needed which is made up of technical people, health managers, health economists and people like me who could be called in for a particular decision. This has to be a supreme body in a manner that nothing can happen in a health department unless a file moves from SHRC (State Health Resource Center). The IAS officers (administrative staff) or principal secretary (politicians) has to implement what the SHRC has decided. Instead of applying their mind, they have to implement what SHRC has decided.

#### Coordination between interventions

In Gujarat, there are several examples of efforts to link interventions on maternal healthcare to each in a way that they support and develop the comprehensive network of interventions needed to provide maternal healthcare. One respondent describes this as:

For example, a doctor is trained in EmOC, simultaneously we train another doctor in anesthesia for maternal healthcare and then a pair would be posted at particular centers so that the can perform caesarian sections together. At the same time there was a missing link of transport. So that missing link was provided through universal number of ambulance, we call it 108 in Gujarat.

The interventions on health system improvements are also linked to interventions aimed at changing the behavior of the public, such as improving eating habits to reduce anemia among pregnant women and information campaigns on the importance of skilled birth attendance. Yet, there are also examples of how coordination remains an important challenge to implementation of interventions of maternal healthcare. One such example is the implementation of First Referral Units.

It takes time to establish, money, lots of training and manpower (to implement the intervention on blood storage at the FRUs). We have planned for blood storage Units in each FRU. To function, blood storage unit requires a certification from competent authority (Food and Drug Control Authority). It also requires a separate room with necessary things (referring to equipment and supplies). Once these criteria are fulfilled, then only the blood store units can function.

To implement strategies that are dependent on several interventions and that require comprehensive coordination between different institutions at different levels of the health system is difficult, and this is perceived as a remaining challenge among the respondents.

### The role of follow-up

#### The monitoring system

Systems to monitor the healthcare provided during pregnancy, delivery and the postnatal period, and the number of maternal deaths, are stressed as important for the implementation process by all respondents of the interviews. Data on figures from the field are stressed as crucial to assess the needs in specific areas of the state and also to continuously follow-up the progress being achieved. Monitoring systems are also seen as key in making evaluations needed to make necessary adjustments. The importance of monitoring as a way of ensuring accountability is also raised. One respondent said that (policies will be implemented when).

it is being monitored and people are accountable for it. A person who is an efficient administrator for that particular program, he keeps on asking questions every time and then people will have to answer him. On the other hand, other people are not asking and no one bothers for it.

In India, it is mandatory to register all births and deaths and maternal deaths can be captured through different systems of recording. In Gujarat, there are several records where maternal mortality is being registered and this is something that is brought up by the respondents as something that is likely to affect the accuracy of the data. One respondent says:

…. the biggest challenge that we are having, and in spite of the help of all professionals and consultants, they just add one or more formats. In spite of collecting and putting the details in one simple format, new formats are implemented and old ones remains as it is. So, that is quite a big issue for us. At the district level, also, we are at the loss to understand the significance that what is the use of so many forms, findings out the use of everything, many things are duplicated in one or the other formats. Despite of doing field work these people are more involved in keeping track of databases.

Another respondent says that

At present, we are filling the same information in 5 to 6 versions of the same software … No officer would like to array the information, which is being submitted to them.

#### Quality of data

The confidence in the quality of the data collected and the ways it is being used to conduct evaluations and follow-up differs between the respondents but most agree that there are important biases in the data due to several difficulties in how data are collected and analyzed.

At present, the weakest part of our system, that I perceive, is our reporting. We are just getting figures, but we are not getting the faces that lay behind these figures.

Another respondent says:

I will be at a loss, because one format will say (give) different information on how many deaths have happened or how many births have taken place. This is the most painful thing, that no one relies on our figures … This is so much hotchpotch. Nobody relies on it. No seniors, no juniors, including myself.

## Discussion

The study of implementation derives from the notion that decisions taken by policymakers may not be carried out as intended (30). To explore possible gaps in the implementation, this study used the policy triangle developed by Walt and Gilson (14). This framework devotes attention not only to the content of the policy, which has been the traditional way of focusing studies of the implementation of health policy, but also the context in which policy is being implemented and to the processes and actors involved. The findings from this study illustrates how the different components of the policy triangle are intertwined to each other, making implementation dependent on content of the policy, the context into which a policy is being implemented, the actors involved and the capacity of the system. Following on from this, the different components are discussed in light of the findings from this study and how these are linked in influencing the capacity of the heath system in Gujarat to implement maternal health policies.

Decision being made and the course of action advocated are found in this study to be perceived as being more dependent on individual actors than on sustainable structures. This is likely to have an impact on long-term memory but potentially also on the motivation of working with a strategy knowing that it might never be implemented. Continuity as an important factor in succeeding in implementing health policy is also found in a study conducted on the health system reform in Pakistan, where a change in heads in the government heads is found to be a trigger likely to lead to a shift in focus and loss of momentum (31).

Findings from this study also indicate that the context in which interventions on maternal health are being implemented into is facing limitations in terms of coordination. The goal of improving maternal health is dependent on many actors at different levels of the health system, and ensuring that adequate maternal health services are available is dependent on a number of interventions. The findings from this study indicate a deficiency in the health system's ability to coordinate the many components needed to improve maternal health in Gujarat remains deficient. Similar results can be found in a comparative study of policy processes in maternal health in India, Vietnam, and China, where coordination in regards to health concerns, such as maternal health, that span across different sectors of health is found to be a key factor in successful implementation (32). The comprehensiveness of the Indian health system is discussed in the World Bank report from 2002, where limited oversight and ability to coordinate health systems components is addressed as one of the most important challenges necessary to overcome to improve the capacity of the Indian health system to implement health policy (33).

Additionally, the findings from this study suggest that the data collected to provide figures on progress on maternal health indicators and service available are not being used to evaluate and develop interventions and that this is perceived as a gap in the system. Both the structure of the system, with several parallel monitoring systems being used, and the confidence in the data being collected are perceived as reasons for this gap. This finding is supported by a study from 2009, showing that limitations in coordination of the monitoring system lead to under-reporting and bias in data available from these systems (34).

Improved maternal health is dependent on many factors in society and is demanding in terms of being dependent on a wide range of health care services needed to ensure complete care (35). Experiences from Malaysia and Sri Lanka, where policy has been successful in achieving reduced maternal mortality, further support the notion that interventions are dependent on the state of the health system (36). Potter and Brough conclude in their study of the Indian health and family welfare sector that ‘In India, there is usually a lack of capacity in terms of structures and processes which allow health workers/managers and facilities to fulfill their potential’ (37). This is further supported by studies done on the reproductive health sector in India (12, 38–40) and in Gujarat (24, 41, 42). The findings from this study reflect the findings from these previous studies, and suggest that limitations in the capacity of the health system cause challenges to the implementation of maternal health interventions in Gujarat.

This study aimed at exploring the process of implementation of maternal health interventions in Gujarat. Since little research has been conducted on this topic, the objective and design of the study were deliberately kept wide. The focus of the study is on overarching structure rather than on single interventions. It is likely that there are variations between interventions and these variations are not captured in this study. The findings from this study suggest limitations in the capacity of the health system to implement interventions on maternal health, and each of these limitations needs to be further explored to be fully understood and described. The objective of this study was to explore the perceptions of high-level stakeholders working with maternal health in Gujarat and the findings are based on the perspective of these. It is likely that the position of these stakeholders in society and in the health system influences the way that they perceive challenges in the process of implementation. It is possible that additional or contradictory challenges would be stressed among stakeholders further down in the health system, and further studies are needed to explore such views to be able to provide a more comprehensive picture of the capacity of the health system.

### Conclusions and policy implications

Maternal mortality and morbidity needs to be addressed with a wide range of interventions and progress is dependent on the capacity of the health system to implement policy. In Gujarat, the health system needs to be improved in terms of the capacity to coordinate interventions. The findings from the study presented in this paper suggests that improvements in coordination between different levels in the health system and between single interventions, strengthening of organizational structure to ensure sustainability and long-term memory, and increased validity and use of data generated through monitoring systems would strengthen the capacity of the health system in Gujarat to implement maternal health policies.
